# An efficient heterogeneous signcryption for smart grid

**DOI:** 10.1371/journal.pone.0208311

**Published:** 2018-12-18

**Authors:** Chunhua Jin, Guanhua Chen, Changhui Yu, Jinsong Shan, Jianyang Zhao, Ying Jin

**Affiliations:** Faculty of Computer and Software Engineering, Huaiyin Institute of Technology, Huai’an, China; Universita degli Studi della Tuscia, ITALY

## Abstract

A smart grid, considered the next-generation type of power grid, combines a traditional power grid with information and communication technologies to effectively facilitate power generation and ensure transmission security and reliability in real-time. Only authorized consumers should be able to access the smart grid because the information gathered by smart meters includes users’ private information. However, smart grid security is still a challenge. Motivated by this challenge, in this paper, we propose a heterogeneous signcryption (HSC) scheme for secure communication between smart meters and the utility. We demonstrate that this scheme is indistinguishable against adaptive chosen-ciphertext attacks (IND-CCA2), existentially unforgeable against adaptive chosen-message attacks (EUF-CMA) and ciphertext-anonymous against adaptive chosen ciphertext attacks (ANON-CCA2) under the computational Diffie-Hellman (CDH) problem in the random oracle model. Our scheme simultaneously achieves confidentiality, integrity, authentication, non-repudiation and ciphertext anonymity in a single logical step. It supports heterogeneous systems, allowing a meter in an identity-based cryptography (IBC) environment to transmit electrical usage data to a utility in a public key infrastructure (PKI) environment. Compared with other existing related schemes, our scheme has the lowest communication overhead and energy consumption for the smart grid. Based on these features, our scheme is highly suitable for secure power transmissions in a smart grid.

## Introduction

Smart grid is envisioned as a next-generation power grid that aims to provide users with electricity in a more reliable and efficient manner [1–5]. The main feature of a traditional power grid is one-way electricity distribution from power plants to consumers. In contrast, a smart grid integrates advanced communication technologies into the traditional grid, allowing two-way energy and information flow. In addition, a smart grid provides consumers with tools to optimize their energy consumption.

Smart meters, which include processors and storage, are key components of a smart grid. Smart meters can communicate with household appliances as well as with facilities at the utility. A smart grid equipped with smart meters can monitor electricity distribution and consumption information in real-time, provide subscribed users with power and fulfill advanced demands as well as manage power usage and outages [6] through a reliable communication network. A smart meter at each home collects electrical usage data from all the electric appliances at the home and transmits these data to the utility company. Thus, a smart grid can provide specific real-time power usage details through the communications between the smart meters and the utility. Then, the utility can change the price of power accordingly. Moreover, it also can adjust users’ power usage using preset load controls to flatten peak demands and avoid potential blackouts. Customers can obtain information about their electricity usage from the smart meters, and thus reschedule their current electric power usage, transferring power usage from peak times to non-peak times to control their costs.

A smart grid provides large benefits for both consumers and the utility. However, its success heavily relies upon communications systems, and the vulnerabilities inherent to communications systems can clearly affect the smart grid, cause severe harm to the entire infrastructure, and damage the economy, the society and affect people’s lives. Thus, communications security is a primary concern in smart grids [7–15]. In this paper, we concentrate primarily on sending power consumption information from smart meters to a utility in a secure manner. The basic considerations are as follows. 1) The power consumption data should be obtainable only by the smart meters and the utility. No other entities should be able to obtain the power consumption data because these data are sensitive. 2) The power consumption data must be authenticated. Without authentication, power consumption data are potentially fake. 3) The power consumption data must not have been altered during transmission. If the power consumption data have been modified, malicious operations have been detected. 4) After a smart meter has sent a consumer’s data to the utility, it cannot retroactively deny its action. 5) The power usage data include no extractable information that can help a third party to identify either the meter or the utility.

It is difficult to propose a scheme that simultaneously meets all the abovementioned properties. Additionally, we must consider that the computational and communication resources of a smart meter are limited. However, the utility has strong computational and communication resources. Thus, the resources available to smart meters and to the utility are not equivalent. Thus, we propose a heterogeneous secure signcryption scheme that accords with such characteristics. The advantage of this heterogeneous scheme is that smart meters have no certificate management problem, but the utility can afford the overhead involved in certificate management.

To ensure secure communications from smart meters to the utility, in this paper, we design a secure HSC scheme. This scheme supports heterogeneous operations on the communication entities. There are three primary innovative points made in this paper.

- First, based on the fact that energy usage data must be well protected, we propose a secure HSC scheme to simultaneously achieve confidentiality, authentication, integrity, non-repudiation and ciphertext anonymity in a logical single step.
- Second, to analyze the security strength of our scheme, a provable security technique is employed to formally prove the proposed scheme’s security. This scheme has the properties of IND-CCA2 [16] and EUF-CMA [16] under the CDH problem in the random oracle model. According to this performance analysis, we conclude that the proposed scheme is more efficient than any other existing HSC schemes [17–19].
- Third, we adopt the heterogeneous communication system. Specifically, we require that a smart meter working in an IBC system be able to send a message to a utility belonging to a PKI system. This heterogeneous characteristic allows our scheme to be used for power information transmission in a smart grid because smart meters have no certificate management ability.

The reminder of the paper is arranged as follows. Related works are reviewed in Section 2. The system model, security requirements, design goal and bilinear pairings are introduced in Section 3. Then, the HSC scheme is designed in Section 4. We discuss its security and performance in Sections 5 and 6, respectively. Finally, Section 7 provides conclusions.

### Related work

Signcryption [20] is a cryptographic primitive that can simultaneously fulfill the functions of a digital signature and public key encryption in a logical single step. Meanwhile, its cost is significantly lower, and its performance exceeds those of the traditional sign-then-encrypt approach. These advantages make signcryption particularly beneficial in environments with limited resources because the properties of confidentiality, authentication, integrity and non-repudiation can be achieved simultaneously at a lower cost. Some PKI-based signcryption schemes [16, 21–23] and some IBC-based signcryption [24–28] schemes have been proposed. But these signcryption schemes are homogeneous; in other words, both the sender and the receiver must be working in the same environment. This requirement of homogeneity is unsuitable for heterogeneous communications.

To employ signcryption in heterogeneous systems, efficient and secure signcryption schemes must be constructed that support heterogeneous communications. Sun and Li [17] presented two HSC schemes. The first HSC scheme permits a sender that belongs to a PKI to transmit a message to a receiver that belongs to an IBC, while the second HSC scheme permits a sender that belongs to an IBC to transmit a message to a receiver that belongs to a PKI. However, these two schemes are not secure from insider attacks because such signcryption schemes have no non-repudiation guarantees. The notion of insider security is stronger than that of outsider security [29], and has two requirements: (1) if the private key of a sender is revealed, an attacker cannot obtain the original message; and (2) if the private key of a receiver is revealed, an attacker cannot counterfeit a ciphertext.

Regarding insider security, Huang et al. [18] presented an HSC scheme that permits a sender who belongs to an IBC to transmit a message to a receiver that belongs to a PKI. This approach is very promising and has triggered considerable followup research [19, 30–32]. For example, Li and Xiong(hereafter called LX) [19] presented a heterogeneous online/offline signcryption (HOOSC) scheme that splits the SC into two phases: an offline phase and an online phase. The offline phase has no knowledge of messages, and most of the complex computations are implemented in this phase. In contrast, the online phase has knowledge of messages and performs only simpler calculations. In 2013, Li et al. [30] presented two SC schemes that support heterogeneous communication. The first HSC permits a sender belonging to a PKI environment to send a message to a receiver belonging to an IBC environment, while the second HSC permits a sender belonging to an IBC environment to send a message to a receiver belonging to the PKI environment. Recently, Li et al.(hereafter termed LZJ) [31] constructed a heterogeneous ring signcryption (HRSC) scheme that works from sensors to servers. The proposed scheme can protect the privacy of the sensor nodes. It permits a sensor node belonging to an IBC environment to send a message to a server belonging to a PKI environment. In 2016, Li et al.(hereafter called LHJ) [32] constructed an HSC scheme intended for communications from wireless sensor networks (WSNs) to an Internet server. In [32], the WSNs belong to a certificateless cryptography environment while the server works in a PKI environment.

### Motivation and contribution

The motivation of this paper is to design a secure heterogeneous signcryption for smart grid. In our scheme, we adopt heterogeneous system which allows smart meters belonging to an IBC environment to transmit electrical usage data to a utility belonging to a PKI environment. The heterogeneity makes our scheme be suited to smart grid. We show that the proposed heterogeneous signcryption is indistinguishable against adaptive chosen-ciphertext attacks (IND-CCA2), existentially unforgeable against adaptive chosen-message attacks (EUF-CMA) and ciphertext-anonymous against adaptive chosen ciphertext attacks (ANON-CCA2) under the computational Diffie-Hellman (CDH) problem in the random oracle model. Our scheme can attain the insider security for confidentiality, integrity, authentication, non-repudiation and ciphertext anonymity in a single logical step. For performance analysis, our scheme has the lowest communication overhead and energy consumption for the smart grid.

## System model, security requirements and design goals

In this section, we describe the system model, security requirements and design goals.

### System model

Our heterogeneous system model, which includes three entity types: a PKG (Private Key Generator), a smart meter and a utility. The PKG is responsible for smart meter registration; it allocates an identity and a corresponding private key to every smart meter. It is always assumed to be trustworthy and never compromised. The smart meter is responsible for collecting electrical usage data and sending the collected data to the utility. The utility is responsible for detecting, responding, adjusting, and storing the power data.

### Security requirements

Security is important for smart grid communications. In our system model, we assume that both the PKG and the certificate authority (CA) are trustable. However, an adversary exists who may eavesdrop or intercept users’ power data and the utility’s management control messages. The adversary may also perform attacks that affect data integrity. Moreover, the smart meters cannot deny any data they have previously transmitted. Therefore, to protect the electrical usage data, a smart grid must satisfy the following security requirements.

- Confidentiality: Power usage information and management control messages should be kept secret to protect consumers’ privacy and the utility’s business information from anyone except the smart meters and the utility.
- Authentication: Only a valid smart meter should be able to send electrical usage data to the utility and receive the corresponding utility services.
- Integrity: The smart grid should be able to ensure that electrical usage data from smart meters and management messages from the utility have not been modified by unauthorized entities.
- Non-repudiation: Once a smart meter has sent electrical usage data to the utility, that action cannot be retroactively denied (i.e., the smart meter cannot deny having transmitted the previous electrical usage data).
- Scalability: Every smart meter sends its electronic data to the utility which realize one to one communication. We add a data collector in the sender to achieve multiple to one communication.

### Design goals

Based on the system model described above and the security requirements, our design goal is to construct an efficient HSC scheme to ensure smart grid security. Specifically, we must achieve the following three objectives.

- Heterogeneous systems could participate in the constructed scheme. As noted above, smart meters have limited computing capacity and storage resources, while the utility has strong computing, energy, bandwidth and storage capacities. Therefore, the proposed scheme should match these characteristics.
- Our proposed scheme should achieve all the security requirements. We know that security is important for smart grids. If security is not ensured, the electricity usage data from the smart meters and the management messages from the utility could conceivably be forged and/or modified by an adversary. Therefore, our constructed scheme should achieve confidentiality, authentication, integrity and non-repudiation simultaneously.
- The proposed scheme should achieve effective communications. Because the power transmission between the smart meter and the utility must meet real-time requirements, our constructed scheme must satisfy the requirements for effective communication.

## Preliminaries

In this section, the bilinear pairings and the CDH problem are outlined.

Let *G*_1_ and *G*_*T*_ be a cyclic additive group and a cyclic multiplicative group. The generator of *G*_1_ is *P*. *G*_1_ and *G*_*T*_ have the same order *q*. A bilinear pairing is a map $\hat{e}:G_{1}\times G_{1}\to G_{T}$ with the following three properties:

1. Bilinear: On inputting $P,Q\in G_{1},a,b\in Z_{q}^{*}$, we have $\hat{e}\left(aP,bQ\right)=\hat{e}{\left(P,Q\right)}^{ab}$.
2. Non-degeneracy: There exists a *P*, *Q* ∈ *G*_1_ such that $\hat{e}\left(P,Q\right)\neq 1$.
3. Computability: On inputting *P*, *Q* ∈ *G*_1_, an efficient algorithm exists to compute $\hat{e}\left(P,Q\right)$.

A bilinear pairing that satisfies the abovementioned properties is called an admissible bilinear pairing. The modified Weil pairing or Tate pairing are admissible maps of this type. For more details, readers can refer to [33].

On inputting a cyclic addition group *G*_1_, its prime order *q* and generator *P*, the CDH problem in *G*_1_ involves computing *abP* given (*P*, *aP*, *bP*) ∈ *G*_1_.

**Definition 1**. The (*ϵ*, *t*)-CDH assumption holds when no *t*-polynomial time adversary A exists who has advantage of at least *ϵ* in solving the CDH problem.

## An HSC scheme

In this section, we first provide the syntax and security notions for an HSC scheme that permits only a sender belonging to an IBC system to transmit a message to a receiver belonging to a PKI system. Here, we employ IP-HSC to denote the following SC, in which “I” denotes IBC and “P” denotes PKI. Then, we describe our proposed HSC scheme.

### Syntax

A generic IP-HSC scheme comprises the following five algorithms.

*Setup*: On inputting a security parameter *k*, this algorithm, which executes on a PKG, outputs a master private key *s* as well as the system parameters *params*.
*IBC-KE*: On inputting the master key *s* and an identity *ID* of a user, this algorithm, which executes on a PKG, outputs a secret key *S*_*ID*_. The PKG securely transmits the secret key to the corresponding user.
*PKI-KG*: This algorithm is executed by PKI users. The user selects a secret key *x* and calculates a corresponding public key *y* which is signed by its CA.
*SC*: On inputting a message *m*, a sender’s secret key $S_{ID_{s}}$ and a receiver’s public key *y*_*r*_, this algorithm (executed by the sender) returns a ciphertext *σ*.
*USC*: On inputting a ciphertext *σ*, the identity *ID*_*s*_ of a sender as well as the receiver’s private key *x*_*r*_, this algorithm (executed by the receiver) returns a message *m* when *σ* is valid or a symbol ⊥ when *σ* is not valid.

For consistency, the algorithm should satisfy the following requirement: if

$$\begin{array}{c} \sigma =Signcrypt(m,S_{ID_{s}},y_{r}) \end{array}$$

then we have

$$\begin{array}{c} m=Usigncrypt(\sigma ,ID_{s},x_{r}) \end{array}$$

### Security notions

Both confidentiality and unforgeability should be satisfied in a signcryption scheme. Here, we slightly amend the notions in [24–26, 34, 35] to adjust IP-HSC.

For confidentiality, the following game is enacted between a challenger C and an adversary F.

*Initial*: On inputting a security parameter *k*, C executes the *Setup* algorithm and sends a master private key *s* as well as the system parameters *params* to the adversary F. Additionally, C also runs the *PKI-KG* algorithm to generate the receiver’s private key *x*_*r*_ and public key *y*_*r*_. It transmits *y*_*r*_ to F.
*Phase 1*: F requests USC queries adaptively. For a USC query, F chooses a ciphertext *σ* as well as the identity *ID*_*s*_ of a sender. C runs *USC*(*σ*, *ID*_*s*_, *x*_*r*_) and transmits the result to F.
*Challenge*: F determines when Phase 1 ends. F produces two equal-length messages, *m*_0_ and *m*_1_, as well as the challenge identity $ID_{s}^{*}$ of a sender. C first runs the *IBC-KE* algorithm to obtain the secret key $S_{ID_{s}}^{*}$. Then, C picks a random bit *β* ∈ {0, 1} and transmits $\sigma^{*}=SC\left(m_{\beta },S_{ID_{s}}^{*},y_{r}\right)$ to F.
*Phase 2*: As in phase 1, F again performs USC queries in an adaptive manner. Nevertheless, it cannot perform a USC query on ($\sigma^{*},ID_{s}^{*},x_{r}$) to obtain the corresponding message this time.
*Guess*: Therefore, F generates a bit *β*′ and wins the game if *β* = *β*′.

F’s advantage is defined as Adv(F) = |2*Pr*[*β*′ = *β*] − 1|, where Pr[*β*′ = *β*] denotes the probability that *β*′ = *β*.

**Definition 2**(Confidentiality). An IP-HSC scheme is (*ϵ*, *t*, *q*_*u*_)-IND-CCA2 secure when no PPT (probabilistic polynomial time) adversary F succeeds with an advantage of at least *ϵ* after at most *q*_*u*_ USC queries.

Notice that the aforementioned definition obtains the confidentiality’s insider security because F is aware of the master private key and all senders’ private keys [29]. This corresponds to the insider security requirements that the signcryption scheme’s forward security must be ensured, and means that confidentiality is maintained even if the sender’s secret key is compromised.

For unforgeability, we consider the following game interacted between a challenger and an adversary F.

*Initial*: On inputting a security parameter *k*, C executes the *Setup* algorithm and transmits the system parameters to F. Additionally, C executes the *PKI-KG* algorithm to obtain the receiver’s private key *x*_*r*_ and public key *y*_*r*_ and transmits them to F.
*Attack*: F requests key extraction queries and signcryption queries adaptively. In a key extraction query, F first chooses an identity *ID* and transmits it to C. Then, C executes the *IBC-KE* algorithm and transmits the corresponding secret key $S_{ID_{s}}$ to F. In a signcryption query, F generates an identity *ID*_*s*_ of a sender as well as a message *m*. C first runs *IBC-KE* algorithm to obtain the private key $S_{ID_{s}}$ of the sender. Then, C sends $\sigma =SC(m,S_{ID_{s}},y_{r})$ to F.
*Forgery*: F generates a challenge identity $ID_{s}^{*}$ of a sender as well as a challenge ciphertext σ*. It succeeds if the following conditions hold:
  1. $USC\left(\sigma^{*},ID_{s}^{*},x_{r}\right)=m^{*}$.
  2. F has not requested a key extraction query on identity $ID_{S}^{*}$.
  3. F has not requested a signcryption query on ($m^{*},ID_{s}^{*}$).

The advantage of F is defined as the probability that it wins.

**Definition 3**(Unforgeability) An IP-HSC scheme is (*ϵ*, *t*, *q*_*k*_, *q*_*s*_)-EUF-CMA secure, if no PPT (probabilistic polynomial time) adversary F succeeds with an advantage of at least *ϵ* after at most *q*_*k*_ key extraction queries and *q*_*s*_ signcryption queries.

In the above definition, note that the adversary is aware of the receiver’s private key *x*_*r*_. This corresponds to the insider security requirement for the unforgeability of a signcryption scheme [29].

### Proposed IP-HSC scheme

In this section, we present an efficient IP-HSC scheme for secure smart grid communications that mainly comprises five algorithms: *Setup*, *IBC-KE*, *PKI-KG*, *SC* and *USC*. Then, we present the design of IP-HSC. We list the main notations of our scheme in Table 1.

**Table 1:** Notations.

Notations	Descriptions
*k*	A security parameter
*G*_1_	A cyclic addition group
*G*_2_	A cyclic multiplicative group
*e*	A bilinear map *e*: *G*_1_ × *G*_1_ → *G*_2_
*P*	A generator of group *G*_1_
*q*	The order of group *G*_1_ and *G*_2_
*n*	The size of a message to be signcrypted
*s*	A master private key of PKG
*P*_*pub*_	A master public key of PKG
*H*_*i*_()	A collision-resistant hash function (*i* = 1, 2, 3)
*ID*_*s*_	An identity of a sender
$Q_{ID_{s}}$	A public key of a sender
$S_{ID_{s}}$	A private key of a sender with identity *ID*_*s*_
*x*_*r*_	A private key of a receiver
*y*_*r*_	A public key of a receiver

*Setup*: On inputting a security parameter *k*, the PKG selects the bilinear map groups (*G*_1_, *G*_2_) of prime order *q*, a generator *P* for *G*_1_ and a bilinear map *G*_1_ × *G*_1_ → *G*_2_. It then chooses a master private key $s\in Z_{q}^{*}$, a master public key *P*_*pub*_ = *sP*, and the hash functions *H*_1_: {0, 1}* → *G*_1_, $H_{2}:{\left\{0,1\right\}}^{n}\times G_{1}^{3}\to G_{1}$, $H_{3}:G_{1}^{3}\to {\left\{0,1\right\}}^{n}$. Here, *n* denotes the size of a message to be signcrypted. The public parameters are {*G*_1_, *G*_2_, *e*, *q*, *P*, *P*_*pub*_, *n*, *H*_1_, *H*_2_, *H*_3_}.
*IBC-KE*: A sender belonging to an IBC transmits its identity *ID*_*s*_ to PKG. The PKG calculates $Q_{ID_{s}}=H_{1}\left(ID_{s}\right)$ and sends the private key $S_{ID_{s}}=sQ_{ID_{s}}$ to the sender.
*PKI-KG*: A receiver in a PKI selects a random value $x_{r}\in Z_{q}^{*}$ as its private key and computes *y*_*r*_ = *x*_*r*_*P* as the corresponding public key.
*SC*: On inputting a message *m*, the sender’s private key $S_{ID_{s}}$, and the receiver’s public key *y*_*r*_, the sender executes the following procedures.
  1. Choose $r\in Z_{q}^{*}$ randomly and compute *U* = *rP*.
  2. Compute *h*_2_ = *H*_2_(*m*, *U*, *ID*_*s*_, *y*_*r*_).
  3. Compute $V=S_{ID_{s}}+rh_{2}$.
  4. Compute *W* = *m* ∥ *ID*_*s*_ ⊕ *H*_3_(*U*, *y*_*r*_, *ry*_*r*_).
  5. Output the ciphertext *σ* = (*U*, *V*, *W*).
*USC*: On inputting a ciphertext *σ*, a sender’s public key $Q_{ID_{s}}$, and a receiver’s private key *x*_*r*_, the receiver executes the following procedures.
  1. Compute *T* = *x*_*r*_*U*.
  2. Compute *m* ∥ *ID*_*s*_ = *W* ⊕ *H*_3_(*U*, *y*_*r*_, *T*).
  3. Compute *h*_2_ = *H*_2_(*m*, *U*, *ID*_*s*_, *y*_*r*_).
  4. Check whether $e\left(P,V\right)=e\left(P_{pub},Q_{ID_{s}}\right)e\left(U,h_{2}\right)$. If so, output the message *m*. Otherwise, reject and output a failure symbol ⊥.

Our IP-HSC scheme is heterogeneous, which is different from HSC [24–26, 34, 35]. In our proposed scheme, the sender is in an IBC system while the receiver is in a PKI system. Therefore, the characteristics of heterogeneous systems are highly suitable for power usage data transmission in a smart grid. A smart meter belonging to the IBC system employs the *SC* algorithm to obtain a ciphertext and transmits it to a utility belonging to the PKI system. Notice that we use the IBC technique in smart meters, which have no certificate management problem; thus, the computational burden of the smart meters is decreased. We employ the PKI technique at the utility, which has no key escrow problem.

In our scheme, every smart meter sends its electronic data to the utility which realize one to one communication. In smart grid, there will be many smart meters to communicate with the utility. Therefore, in order to achieve scalability, we add a data collector in the sender, which collect data from lots of smart meters. The utility does not need to establish a single communication channel to each smart meter. Thus, we can achieve multiple to one communication. To realize efficiency, the limited computation ability of smart meter does not perform many expensive calculation.

## Security analysis

In this section, we analyze the confidentiality and unforgeability of our proposed IP-HSC scheme by following Theorem 1 and 2, respectively.

**Theorem 1** (Confidentiality) In the random oracle model, if an adversary F exists that can break the IND-CCA2 security of our proposed IP-HSC scheme with a nonnegligible advantage *ϵ*, running in a given time *t* and making at most *q*_*u*_ unsigncryption queries and $q_{H_{i}}$ oracle *H*_*i*_ (*i* = 1, 2, 3) queries, then there exists a PPT algorithm C that settles the CDH problem with an advantage

$$\begin{array}{c} \epsilon^{\prime }>\epsilon \left(1-\frac{q_{u}}{2^{k}}\right) \end{array}$$

in a given time $t^{\prime }<t+O\left(q_{H_{3}}+q_{u}\right)t_{e}$, where *t*_*e*_ is the time of a pairing operation.

*Proof*: It is assumed that we construct an algorithm C that employs F as a subroutine to settle the random instance (*P*, *aP*, *bP*) of the CDH problem.
*Initial*: C randomly selects a master private key *s* and calculates a master public key *P*_*pub*_ = *sP*. C also calculates a receiver’s public key *y*_*r*_ = *aP*. Here *a* simulates the receiver’s private key, and C is not aware of the value of *a*.
*Phase 1*: C acts as the challenger to F in the confidentiality game defined in Section 4. Three lists are kept to simulate the hash oracles *H*_1_, *H*_2_, *H*_3_, respectively. Assume that *H*_1_ queries are distinct. We also assume that F will issue an *H*_1_(*ID*) query before employing *ID* in any other queries.
  - *H*_1_ queries: For an *H*_1_ query on the identity *ID*_*i*_, C first examines whether *H*_1_’s value is already in the list *L*_1_. If yes, the existing value is returned; otherwise, C selects $t_{i}\in Z_{p}^{*}$ randomly, set *t*_*i*_*P* as the value and inserts the tuple (*ID*_*i*_, *t*_*i*_) into the list *L*_1_.
  - *H*_2_ queries: For an *H*_2_ query on (*m*, *U*, *ID*_*s*_, *y*_*r*_), C first determines whether *H*_2_’s value is already in the list *L*_2_. If so, the existing value is returned; otherwise, C picks a random value $e_{i}\in Z_{p}^{*}$, sets *e*_*i*_*P* as the answer and inserts the tuple (*m*, *U*, *ID*_*s*_, *y*_*r*_, *e*_*i*_*P*) into the list *L*_2_.
  - *H*_3_ queries: For an *H*_3_ query on (*U*, *y*_*r*_, *T*), C performs the following steps.
    1. If *e*(*aP*, *bP*) = *e*(*T*, *P*), C outputs *T* and stops. On this occasion, C has settled the given CDH problem.
    2. If a tuple of the form (*U*, *y*_*r*_, *, *h*_3,*i*_) exists in list *L*_3_ such that *e*(*U*, *y*_*r*_) = *e*(*T*, *P*), C outputs *h*_3,*i*_ and regenerates the symbol * with *T*.
    3. If C reaches the execution point, it selects *h*_3,*i*_ ∈ {0, 1}^*n*^ randomly and gives it to F. Then, C saves the query and inserts the response into the list *L*_3_.
*Unsigncryption queries*: F selects a sender’s identity *ID*_*s*_ and a ciphertext *σ* = (*U*, *W*). C performs the following steps.
  1. C searches for a tuple of the form (*U*, *y*_*r*_, *T*) for different *T* values, such that *e*(*U*, *y*_*r*_) = *e*(*T*, *P*). If such an entry exists, *h*_3,*i*_’s correct value can be obtained, and C employs this value *h*_3,*i*_ to decrypt the ciphertext (i.e., *m* = *W* ⊕ *h*_3_). If no such entry exists in *L*_3_, C randomly selects *h*_3,*i*_ ∈ {0, 1}^*n*^ and adds the tuple (*U*, *y*_*r*_, *, *h*_3,*i*_) to the list *L*_3_. Then, C decrypts the ciphertext using the random value *h*_3,*i*_.
  2. C asks an *H*_2_ query and obtains *h*_2,*i*_ = *H*_2_(*m*, *U*, *ID*_*s*_, *y*_*r*_). Then, it checks whether $e\left(P,V\right)=e\left(P_{pub},Q_{ID_{s}}\right)e\left(U,h_{2}\right)$. When the conditions hold, message *m* is returned to F. Otherwise, C rejects the ciphertext.
*Challenge*: F produces two equal length plaintexts (*m*_0_*andm*_1_) and a challenge identity $ID_{s}^{*}$ of a sender. In response, C first sets U* = *bP* and selects W* from {0, 1}^*n*^. Then, C transfers the ciphertext σ* = (U*, W*) to F.
*Phase 2*: F adaptively performs an unsigncryption query again as in Phase 1. There is a restriction that F cannot issue an unsigncryption query on ($\sigma^{*},ID_{s}^{*},x_{r}$) to obtain the corresponding plaintext. C replies to F’s queries following the same approach as in Phase 1.
*Guess*: F generates a bit *β*′ that is neglected by C.

The simulation is perfect except that F requests an *H*_3_ query on the entry (u*, *y*_*r*_, aT*). If no such entry exists in the list *L*_3_, F has no advantage. Nevertheless, if that happens, because of the first step in *H*_3_’s simulation, C will solve the CDH problem. Throughout this entire simulation, the failure probability for unsigncryption queries is at most *q*_*u*_/2^*k*^.

**Theorem 2** (Unforgeability) Under the random oracle model, if an adversary F exists that can break the EUF-CMA security of our proposed IP-HSC scheme, running in a given time *t* and making at most *q*_*k*_ key extraction queries, *q*_*s*_ signcryption queries, and $q_{H_{i}}$ oracle *H*_*i*_ (*i* = 1, 2, 3) queries with a nonnegligible advantage *ϵ*, then there exists an algorithm C that settles the CDH problem with an advantage

$$\begin{array}{c} \epsilon^{\prime }\geq \epsilon \frac{1}{e(q_{k}+1)}\left(1-\frac{q_{s}\left(q_{s}+q_{H_{2}}\right)}{2^{k}}\right) \end{array}$$

in a time of *O*(*t*).

*Proof*: Assume that we construct an algorithm C that employs F as a subroutine to solve the random instance (*P*, *aP*, *bP*) of the CDH problem.
*Initial*: C randomly selects a receiver’s secret key *x*_*r*_ from $Z_{p}^{*}$ and calculates the corresponding public key *y*_*r*_ = *x*_*r*_*P*. Then, C sends the receiver’s key pair (*x*_*r*_, *y*_*r*_) and the system parameters *params* with *P*_*pub*_ = *aP* to F. Notice that C is not aware of the *a* value that simulates the PKG’s master private key.
*Attack*: C acts as the challenger to F in the unforgeability game defined in Section 4. Three lists are kept to simulate the hash oracles *H*_1_, *H*_2_, *andH*_3_. It is assumed that *H*_1_ queries are distinct. We also assume that F will re-query *H*_1_(*ID*) before utilizing *ID* in any other queries.
  - *H*_1_ queries: F performs *H*_1_ queries on identity *ID*_*i*_, as in the proof technique by Coron [36]. C spins a coin *T* ∈ {0, 1} that takes a value of 0 with the probability of *ξ* and a value of 1 with the probability 1 − *ξ*. If *T* = 0, then C picks *n*_*i*_ from $Z_{q}^{*}$ and defines *H*_1_(*ID*_*i*_) = *n*_*i*_*P*. If *T* = 1, then C outputs *H*_1_(*ID*_*i*_) = *n*_*i*_*bP*. In these two cases, C adds a triple (*ID*_*i*_, *n*_*i*_, *T*) to the list *L*_1_.
  - *H*_2_ queries: For an *H*_2_(*m*, *U*, *ID*_*s*_, *y*_*r*_) query, C first examines whether the *H*_2_ value is already in list *L*_2_ for the entry (*m*, *U*, *ID*_*s*_, *y*_*r*_). If so, it outputs the existing value; otherwise, C outputs *h*_2,*i*_ from *G*_1_ as the answer. Then, C inserts the tuple (*m*, *U*, *ID*_*s*_, *y*_*r*_, *h*_2,*i*_) into list *L*_2_.
  - *H*_3_ queries: For an *H*_3_(*U*, *y*_*r*_, *T*) query, C first determines whether the *H*_3_ value is already in list *L*_3_ for the entry (*U*, *y*_*r*_, *T*). If so, it returns the existing value; otherwise, C outputs a random value *h*_3,*i*_ from {0, 1}^*n*^ as the answer. Then, C inserts the tuple (*U*, *y*_*r*_, *T*, *h*_3,*i*_) into the list *L*_3_.
  - *Key extraction queries*: When F performs a key extraction query on an identity *ID*_*i*_, C obtains the corresponding triple (*ID*_*i*_, *n*_*i*_, *T*) from list *L*_1_. When *T* = 1, C fails and stops because it cannot compute the private key. Otherwise, C outputs the private key *n*_*i*_*aP*.
  - *Signcryption queries*: F selects a message *m* and a sender’s identity *ID*_*s*_. In response, C performs the following steps.
    1. Select $r,t\in Z_{q}^{*}$ randomly and compute *U* = *tP*_*pub*_, *V* = *rP*_*pub*_.
    2. Set $t^{-1}\left(rP-Q_{ID_{s}}\right)=H_{2}\left(m,U,ID_{s},y_{r}\right)$ and add the tuple (*m*, *U*, *ID*_*s*_, *y*_*r*_) to the list *L*_2_.
    3. Define *h*_3_ = *H*_3_(*U*, *y*_*r*_, *T*) and insert the tuple (*U*, *y*_*r*_, *T*) into the list *L*_3_.
    4. Compute *W* = *m* ⊕ *h*_3_.
    5. Return the ciphertext *σ* = (*U*, *W*).

Eventually, F outputs a challenge ciphertext σ* = (U*, W*) and a challenge identity $ID_{s}^{*}$ of a sender. Then, C retrieves the tuple $\left(ID_{s}^{*},n_{i}^{*},T^{*}\right)$ from the list *L*_1_. If T* = 0, C fails and stops. Otherwise, it continues and list *L*_2_ must contain an item $\left(m^{*},U^{*},ID_{s}^{*},y_{r}^{*},e_{i}^{*}\right)$ with an overwhelming probability. Because $h_{2}^{*}=H_{2}\left(m^{*},U^{*},ID_{s}^{*},y_{r}^{*}\right)$ was defined as $e_{i}^{*}P\in G_{1}$, if F succeeds in the game, C realizes that $e\left(P,V^{*}\right)=e\left(P_{pub},Q_{ID_{s}}^{*}\right)e\left(U^{*},h_{2}^{*}\right)$ with $h_{2,i}^{*}=e_{i}^{*}P$, $Q_{ID_{s}}^{*}=n_{i}^{*}bP$ for $e_{i}^{*},n_{i}^{*}\in Z_{q}^{*}$. Then, C is aware of that $e\left(P,V^{*}\right)=e\left(aP,n_{i}^{*}bp\right)e\left(U^{*},e_{i}^{*}P\right)$ and that $n_{i}^{*-1}\left(V^{*}-e_{i}^{*}U^{*}\right)$ is the solution of the CDH problem.

Now we evaluate the *ρ* value. C’s successful probability in all key extraction queries is at most $\rho^{q_{k}}$. During the forgery phase, the probability that F has not asked a key extraction query for an identity $ID_{s}^{*}$ is 1 − *ρ*. In addition, C’s probability of success for all key extraction queries is $\rho^{q_{k}}\left(1-\rho \right)$. The value is maximized at *ρ*′ = *q*_*k*_/(*q*_*k*_ + 1). Utilizing this value, *ρ*′, we obtain

$$\begin{array}{c} {\left(\frac{q_{k}}{q_{k}+1}\right)}^{q_{k}}\left(1-\frac{q_{k}}{q_{k}+1}\right)=\frac{1}{{\left(1+\frac{1}{q_{k}}\right)}^{q_{k}}}\frac{1}{q_{k}+1} \end{array}.$$

Additionally, utilizing the result lim_λ→0_(1 + λ)^1/λ^ = *e*, we have $\frac{1}{{\left(1+\frac{1}{q_{k}}\right)}^{q_{k}}}\geq \frac{1}{e}$ for large *q*_*k*_ values. Hence, the probability that C will succeeds in key extraction queries is at most $\frac{1}{e(q_{k}+1)}$, while the probability of C failing at all signcryption queries is is $q_{s}\left(q_{s}+q_{H_{2}}\right)/2^{k}$ because a conflict exists on *H*_2_. Therefore, we obtain

$$\begin{array}{c} \epsilon^{\prime }\geq \epsilon \frac{1}{e(q_{k}+1)}\left(1-\frac{q_{s}\left(q_{s}+q_{H_{2}}\right)}{2^{k}}\right) \end{array}.$$

## Performance evaluation

Table 2 shows the performance of the proposed scheme, which is evaluated based on comparing the major computational cost, security, and communication overhead of our scheme with those of existing schemes SL-II [17], HWY-I [18], HWY-II [18] and LX-II [19], which are representative HSC schemes. In these four schemes, the senders work in the IBC setting and the receivers work in the PKI setting. They are denoted by PM, E, PC, the point multiplication in *G*_1_, the exponentiation, and the pairing operation in *G*_2_. Since hash function operation and XOR operations are much cheaper than PM or PC, we ignore those two operations. We assume that the sender in an IBC system has limited computation and storage capability but that the receiver in the PKI system has sufficient computation and storage resources. Therefore, we compare only the computational cost for signcryption. From Table 2, we can see that the computational cost of signcryption in these five schemes is considerable. In the “security” column, CCA2, CMA, and IS, denote IND-CCA2, EUF-CMA, and insider security, respectively. we can see that SL-II [17] does not meet CMA and IS security requirements. HWY-I [18], HWY-II [18], LX [19] and our scheme meet the requirements of insider security. In the “Communication overhead” column, our scheme is the shortest at 432 bits.

**Table 2:** Performance comparison.

Schemes	Computational cost		Security				Communication overhead (bits)
	Signcrypt	Unsigncrypt	CCA2	CMA	ANON	IS	
SL-II [17]	1PC	1PC+1E	Yes	No	No	No	560
HWY-I [18]	3PM	2PM+2PC	Yes	Yes	No	Yes	1328
HWY-II [18]	2PM	4PM	Yes	Yes	No	Yes	1328
LX-II [19]	2PM+1E	2PC+1PM+1E	Yes	Yes	No	Yes	704
Ours	3PM	1PM+3PC	Yes	Yes	Yes	Yes	432

**Fig 1:**
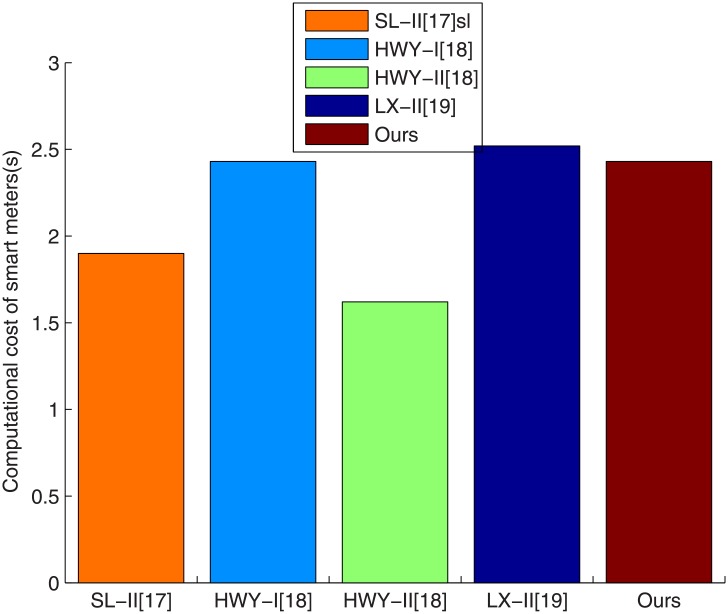
The computational cost of smart meters versus related protocols.

Here we give a quantitative analysis for SL-II [17], HWY-I [18], HWY-II [18], LX-II [19] and our scheme. We also only consider the smart meter part which has limited capacity. The experiment in [37] is adopted on MICA2 which is equipped with an ATmega128 8-bit processor clocked at 7.3728 MHz, 4 KB RAM and 128 KB ROM. According to [37], a PC needs 1.9s and an E needs 0.9s utilizing the supersingular curve *y*^2^ + *y* = *x*^3^ + *x* with an embedding degree 4 and implementing *η*_*T*_ pairing: *E*(*F*_2^271^_) × *E*(*F*_2^271^_)→*F*_2^4⋅271^_ at an 80-bit security level. From [38], a PM operation in the extension field *F*_2^4⋅271^_ takes about 0.81s. As in [37, 38], we can see that the computational time on the meter of SL-II [17], HWY-I [18], HWY-II [18], LX-II [19] and our scheme are 1 * 1.9 = 1.9s, 3 * 0.81 = 2.43s, 2 * 0.81 = 1.62s, 2 * 0.81 + 1 * 0.9 = 2.52s and 3 * 0.81 = 2.43s, respectively. Fig 1 shows the relationship between the computational cost of smart meters and the related protocols. From Fig 1, we can see that the computational cost of our scheme is not the least, which is lower than LX-II [19], but higher than SL-II [17] and HWY-II [18].

According to [37, 39], let us suppose that the current draw in active mode is 8.0mA, the current draw in receiving mode is 10mA, the current draw in transmitting mode is 27mA, the power level of MICA2 is 3.0V, and the data rate is 12.4kbps. For energy consumption, as in [40, 41], a PC operation consumers 3.0 * 8.0 * 1.9 = 45.6mJ, an E operation in *G*_2_ consumers 3.0 * 8.0 * 0.9 = 21.6mJ and a PM consumers 3.0 * 8.0 * 0.81 = 19.44mJ. Hence, the computational energy cost on the meter of SL-II [17], HWY-I [18], HWY-II [18], LX-II [19] and our scheme are 1.9 * 45.6 = 86.64mJ, 3 * 0.81 * 19.44 = 47.24mJ, 2 * 0.81 * 19.44 = 31.49mJ, 2 * 0.81 * 21.16 + 0.9 * 19.44 = 51.78mJ and 3 * 0.81 * 19.44 = 47.24mJ, respectively.

For the communication cost, let us suppose that |*ID*| = 80bits as well as |*m*| = 160bits. Because we employ a subgroup *G*_1_ of the 252-bit prime order, which is based on the supersingular curve *y*^2^ + *y* = *x*^3^ + *x* over *F*_2^271^_, an element’s size in group *G*_1_ is 542bits and can be reduced to 272bits (34 bytes) by means of standard compression technique [37] and an element’s size in group *G*_2_ is 1084bits. Therefore, the meter in SL-II [17], HWY-I [18], HWY-II [18], LX-II [19] and the proposed scheme needs to transmit 560bits = 70bytes, 1328bits = 166bytes, 1328bits = 166bytes, 704bits = 88bytes and 432bits = 54bytes messages. From [37], we can see that the meter consumers 3 * 27 * 8/12400 = 0.052mJ to transmit one byte messages. Hence, the communication energy consumption of the meter in SL-II [17], HWY-I [18], HWY-II [18], LX-II [19] and our scheme are 0.025 * 70 = 1.75mJ, 0.025 * 166 = 4.15mJ, 0.025 * 166 = 4.15mJ, 0.025 * 88 = 2.2mJ, 0.025 * 54 = 1.35mJ. Therefore, the total energy consumption of SL-II [17], HWY-I [18], HWY-II [18], LX-II [19] and our scheme are 86.84 + 1.75 = 88.39mJ, 47.24 + 4.15 = 51.39mJ, 31.49 + 4.15 = 35.64mJ, 51.78 + 2.2 = 53.98mJ and 47.24 + 1.35 = 48.59mJ.

**Fig 2:**
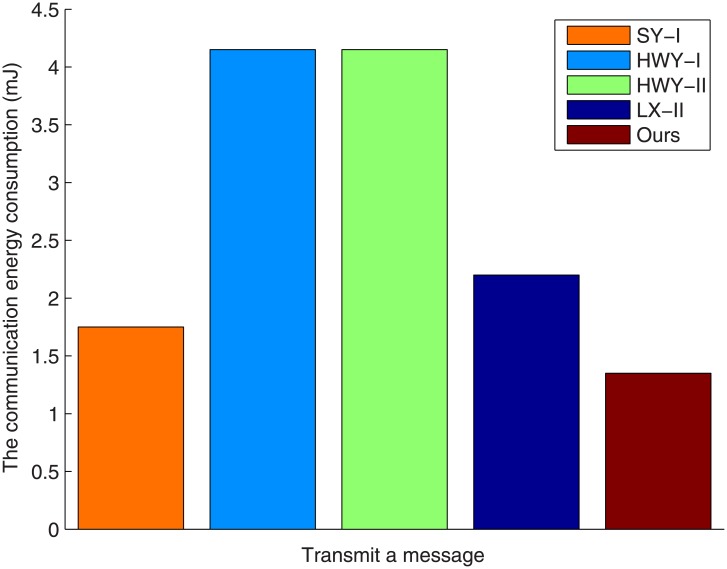
The communication energy consumption versus transmit a message.

The communication energy consumption at the meter is summarized in Fig 2, from which we can see that the proposed scheme requires the least energy consumption for communication among the five tested schemes. We can also see that the proposed scheme needs only 1.35mJ to transmit a message. This energy cost is highly suitable for practical use in a smart grid.

## Conclusion

In this paper, we proposed an efficient HSC scheme for secure smart grid communications that allows a sender to belong to an IBC environment but to transmit a message to a receiver belonging to a PKI environment. The proposed scheme is proved to have IND-CCA2 as well as EUF-CMA properties under the CDH problem in the random oracle model, and it achieves confidentiality, integrity, authentication and non-repudiation simultaneously in a single logical step. Compared with existing HSC schemes that support a sender working in an IBC setting and a receiver working in a PKI setting, our scheme greatly enhances the communication efficiency, which meets the demand for real-time power usage data transmission in smart grid communications. A performance analysis is provided to demonstrate the efficiency improvement.

## Supporting information

S1 Fig The computational cost of smart meters versus related protocols. (PDF)

S2 Fig The communication energy consumption versus transmit a message. (PDF)

S1 File The minimal underlying data set. (DOCX)
