# Supplementary figures and images for: An efficient heterogeneous signcryption for smart grid
Source: PLoS One. 2018 Dec 18;13(12):e0208311. doi: 10.1371/journal.pone.0208311 (PMC6298654; doi:10.1371/journal.pone.0208311)

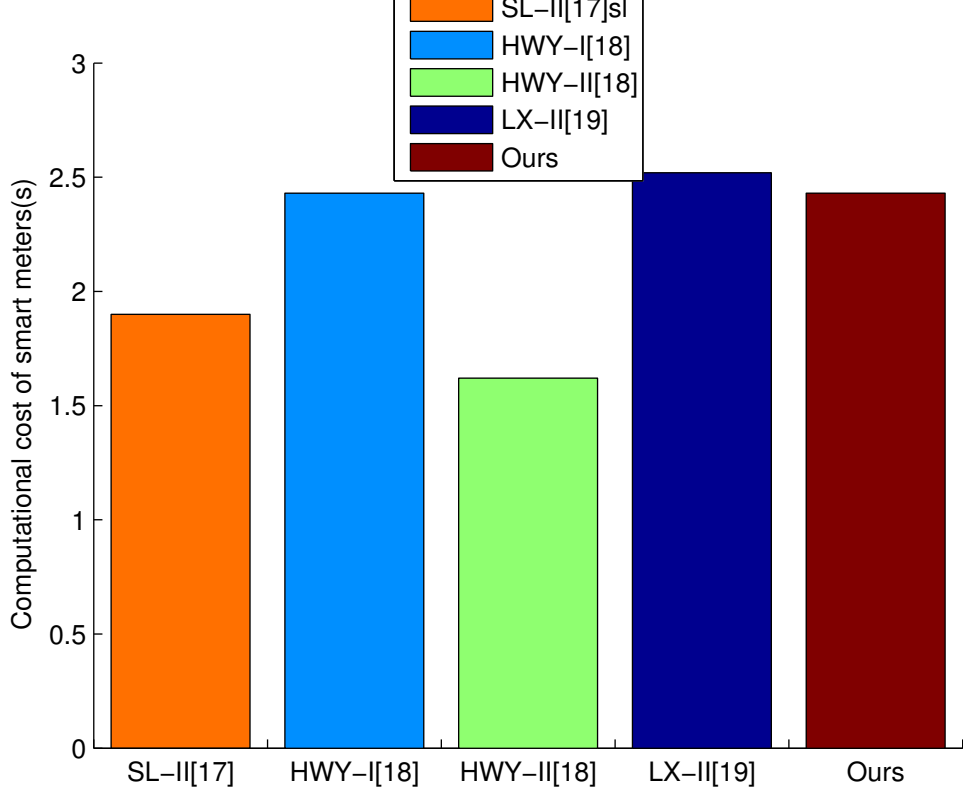

Supplement: S1 Fig — (PDF) [file pone.0208311.s001.pdf]

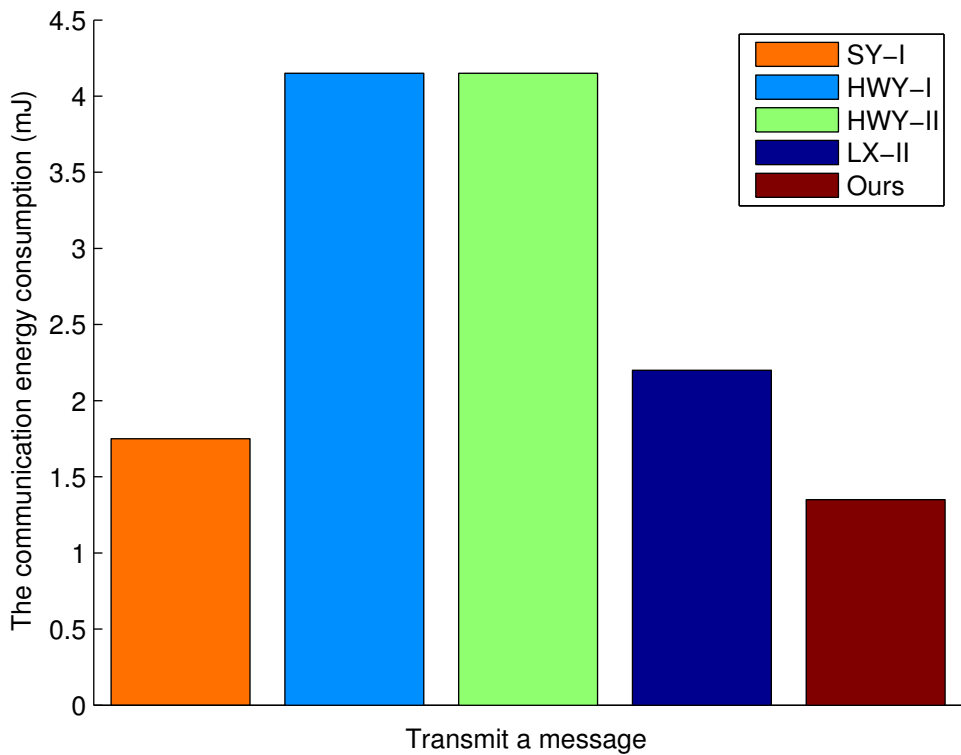

Supplement: S2 Fig — (PDF) [file pone.0208311.s002.pdf]
