# Supplementary material for: An efficient heterogeneous signcryption for smart grid
Source: PLoS One. 2018 Dec 18;13(12):e0208311. doi: 10.1371/journal.pone.0208311 (PMC6298654; doi:10.1371/journal.pone.0208311)
Supplement: S1 File — (DOCX) [file pone.0208311.s003.docx]

In these four schemes, the senders work in the IBC setting and the receivers work in the PKI setting. They are denoted by PM, E, PC, the point multiplication in $G_{1}$, the exponentiation, and the pairing operation in $G_{2}$. Since hash function operation and XOR operations are much cheaper than PM or PC, we ignore those two operations. We assume that the sender in an IBC system has limited computation and storage capability but that the receiver in the PKI system has sufficient computation and storage resources. Therefore, we compare only the computational cost for signcryption.

The experiment is adopted on MICA2 which is equipped with an ATmega128 8-bit processor clocked at 7.3728 MHz, 4 KB RAM and 128 KB ROM. A PC needs 1.9s and an E needs 0.9s utilizing the supersingular curve with an embedding degree 4 and implementing pairing: at an 80-bit security level. From[38], a PM operation in the extension field takes about 0.81s. As in[37,38], we can see that the computational time on the meter of SL-II[17], HWY-I[18], HWY-II[18], LX-II[19] and our scheme are , s, s, s and s, respectively. Figure 3 shows the relationship between the computational cost of smart meters and the related protocols. From figure 3, we can see that the computational cost of our scheme is not the least, which is lower than LX-II[19], but higher than SL-II[17] and HWY-II[18].According to[37,38], let us suppose that the current draw in active mode is 8.0mA, the current draw in receiving mode is 10mA, the current draw in transmitting mode is 27mA, the power level of MICA2 is 3.0V, and the data rate is 12.4kbps. For energy consumption, as in[40,41], a PC operation consumers mJ, an E operation in consumers mJ and a PM consumers mJ. Hence, the computational energy cost on the meter of SL-II[17], HWY-I[18], HWY-II[18], LX-II[19] and our scheme are mJ, $3*0.81*19.44=47.24$mJ, mJ, mJ and mJ, respectively.

For the communication cost, let us suppose that =80bits as well as = 160bits. Because we employ a subgroup of the 252-bit prime order, which is based on the supersingular curve over , an element's size in group is 542bits and can be reduced to 272bits (34 bytes) by means of standard compression technique[37] and an element's size in group is 1084bits. Therefore, the meter in SL-II[17], HWY-I[18], HWY-II[18], LX-II[19] and the proposed scheme needs to transmit 560bits=70bytes, 1328bits=166bytes, 1328bits=166bytes, 704bits=88bytes and 432bits=54bytes messages. From[37], we can see that the meter consumers $3*27*8/12400=0.052$mJ to transmit one byte messages. Hence, the communication energy consumption of the meter in SL-II[17], HWY-I[18], HWY-II[18], LX-II[19] and our scheme are mJ, mJ, mJ, mJ, mJ. Therefore, the total energy consumption of SL-II[17], HWY-I[18], HWY-II[18], LX-II[19] and our scheme are 86.84+1.75=88.39mJ, 47.24+4.15=51.39mJ, 31.49+4.15=35.64mJ, 51.78+2.2=53.98mJ and 47.24+1.35=48.59mJ.
